# Association between fibrinogen/albumin ratio and arterial stiffness in patients with type 2 diabetes: A cross-sectional study

**DOI:** 10.3389/fphar.2022.1120043

**Published:** 2023-01-12

**Authors:** Chun-mei Chen, Chun-feng Lu, Wang-shu Liu, Zhen-hua Gong, Xue-qin Wang, Feng Xu, Jian-feng Ji, Xing-xing Fang

**Affiliations:** ^1^ Department of Geriatrics, Affiliated Hospital 2 of Nantong University and First People’s Hospital of Nantong City, Nantong, China; ^2^ Department of Endocrinology, Affiliated Hospital 2 of Nantong University and First People’s Hospital of Nantong City, Nantong, China; ^3^ Department of Burn and Plastic Surgery, Affiliated Hospital 2 of Nantong University and First People’s Hospital of Nantong City, Nantong, China; ^4^ Department of Nephrology, Affiliated Hospital 2 of Nantong University and First People’s Hospital of Nantong City, Nantong, China

**Keywords:** type 2 diabetes, fibrinogen/albumin ratio, brachial-ankle pulse wave velocity, inflammation, coagulation

## Abstract

**Background:** Fibrinogen albumin ratio (FAR) is significantly correlated with the severity and prognosis of cardiovascular disease (CVD). Arterial stiffness is an early lesion of CVD, but no studies have examined the correlation between arterial stiffness and FAR. This study aimed to examine the relationship between FAR and arterial stiffness in patients with type 2 diabetes (T2D), as measured by brachial-ankle pulse wave velocity (baPWV).

**Methods:** In this cross-sectional investigation, patients with T2D were enrolled between January 2021 and April 2022. In each patient, the levels of fibrinogen and albumin in the serum, and baPWV in the serum were measured. A baPWV greater than 1800 cm/s was utilized to diagnose arterial stiffness.

**Results:** The study included 413 T2D patients. The mean age of these participants was 52.56 ± 11.53 years, 60.8% of them were male, and 18.6% of them had arterial stiffness. There were significant differences in baPWV level and proportion of arterial stiffness (*p* < .001) between the four subgroups categorized by the FAR quartile. The relationships between the FAR and baPWV and arterial stiffness were significantly favorable in the overall population and subgroups of elderly men and non-elderly men (*p* < .01), while they were insignificant in subgroups of elderly and non-elderly women (*p* > .05). To investigate the correlation between the FAR and baPWV, the arterial stiffness and the FAR in male T2D patients, respectively, multivariable logistic regression analysis and multiple linear regression analysis were developed. The lnFAR and lnbaPWV had a significant relationship in the multiple linear regression analysis fully adjusted model. After adjusting for potential covariables, multivariable logistic regression analysis revealed that the FAR was independently associated with arterial stiffness [OR (95% CI), 1.075 (1.031–1.120)]. In addition, receiver operating characteristic analysis indicated that the best FAR cutoff value for detecting arterial stiffness in male T2D patients was 76.67 mg/g.

**Conclusion:** The level of FAR had an independent and positive correlation with baPWV and arterial stiffness in male patients with T2D, but not in female patients.

## Introduction

The rate of type 2 diabetes (T2D) is rising each year, posing a significant threat to public health, as a result of China’s rapid economic development, lifestyle changes, and ageing population (Li et al., 2020). In 2015, cardiovascular problems accounted for the majority of the approximately five million fatalities attributed to T2D and associated consequences worldwide (Zheng et al., 2018). Consequently, the management of cardiovascular disease (CVD) is a crucial aspect of the treatment of T2D. Arterial stiffness, or decreased arterial elasticity, is the earliest lesion of CVD (Zhang et al., 2020). Brachial-ankle pulse wave velocity (baPWV), a reproducible, non-invasive, and easy quantitative measurement of arterial elasticity, is a significant predictor of CVD, myocardial damage, and cardiovascular events (Sheng et al., 2014). Thus, identifying early screening markers of elevated baPWV and initiating early intervention may be advantageous for enhancing cardiovascular outcomes in T2D patients.

Both fibrinogen and albumin are produced in the liver; fibrinogen is implicated in inflammation and clotting cascades (Sörensen et al., 2011), whereas albumin has an inhibitory function in inflammation, platelet activation, and aggregation (Deveci and Gazi, 2021). Albumin and fibrinogen are useful biomarkers of inflammation and hemodynamic changes, respectively. The Fibrinogen/Albumin Ratio (FAR), which combines the two indicators, was more effective than any single indicator in predicting the prognosis of multiple tumors (Geer and Shen, 2009; Yang et al., 2014; Wen et al., 2015). Moreover, FAR performed better than fibrinogen and albumin in determining the severity of acute myocardial infarction (AMI) and predicting the short-term prognosis of patients (Erdoğan et al., 2016; Zhao et al., 2019). A prospective cohort investigation conducted in China revealed that CVD patients with high FAR levels and diabetes had a poorer 5-year prognosis (Wang et al., 2022). The role of FAR in assessing CVD had been thoroughly established in earlier studies, but no investigation had looked at the connection between FAR and the early lesion of CVD, arterial stiffness. Evaluation of FAR will assist in managing CVD throughout T2D if the association between FAR and arterial stiffness in T2D patients is found to exist.

Therefore, this observational study was carried out to investigate the relationship between FAR and baPWV in T2D patients.

## Methods

### Study participants

**FIGURE 1:**
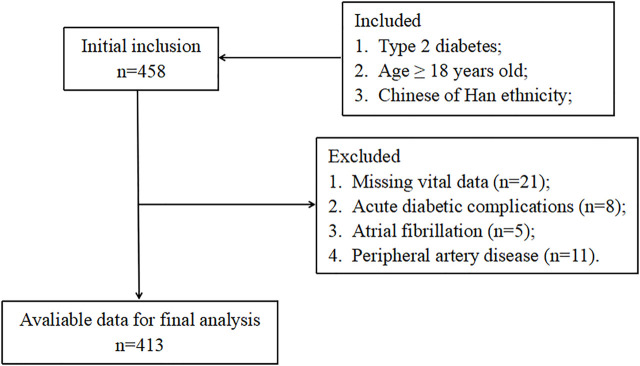
The study flowchart.

The current study included T2D patients who were hospitalized in the endocrinology department of the second affiliated hospital of Nantong University from January 2021 to April 2022. T2D was diagnosed using the American Diabetes Association criteria (American Diabetes Association, 2014). Exclusion criteria included any one or more of the following: type 1 diabetes, acute diabetic complications, receiving steroid therapy, taking any anticoagulant, malignant diseases, chronic hepatopathy, atrial fibrillation, peripheral artery disease, heart failure, acute infections, blood diseases, and autoimmune diseases. Figure 1 exhibits the detailed study flowchart. In total, 413 T2D patients were ultimately included in this study. Each participant provided written, informed consent and the study was conducted following the Declaration of Helsinki.

### Basic data collection

Checking medical records allowed for the collection of information on demographics, drinking and smoking habits, past medication usage, and disease diagnoses. The medication history was particularly focused on the use of drugs for T2D, hypertension, and dyslipidemia.

### Anthropometric assessments

Each subject was measured for anthropometric characteristics, including weight, height, and blood pressure. Body mass index (BMI) was determined by the following formula: weight (kg)/height (m^2^). The average of the two blood pressure readings was recorded for further analysis after being measured twice with a standard mercury sphygmomanometer.

### Laboratory examinations

After a 12-h overnight fast, samples of blood and fresh first-void morning urine were obtained. FAR was determined by dividing the serum fibrinogen level by the serum albumin level after measuring the levels of serum fibrinogen and albumin. The estimated glomerular filtration rate (eGFR) was computed utilizing the CKD-EPI creatinine-cystatin C equation (2012) (Inker et al., 2012).

### Brachial-ankle pulse wave velocity (baPWV)

As previously described, each subject’s baPWV was measured by a trained technician utilizing an automated system (BP-203RPE III device, Omron, Japan) (Wu et al., 2019). Each subject lay down for detection in the supine position after at least five minutes of rest. For analysis, the baPWV value with the higher difference between the right and left sides was selected. A baPWV ≥ 1800 cm/s was utilized to define arterial stiffness (Takashima et al., 2014).

### Statistical analyses

SPSS statistical software version 18.0 (IBM SPSS Inc., United States) was utilized for the analysis of the data. A *p* value < .05 was considered as statistical significance. Continuous variables with skewed and normal distributions and categorical variables were presented as the mean ± standard deviation (SD), frequencies with percentages, and medians with interquartile range, respectively. For analysis, the data on fibrinogen and albumin levels were ln-transformed due to their skewed distributions. Comparisons of continuous variables with skewed and normal distributions and categorical variables were conducted between groups using the Kruskal–Wallis test and the one-way analysis of variance, and the chi-square test, respectively. Spearman’s analyses of bivariate correlation were applied to measure the strength of associations between FAR level and other clinical variables. In addition, spearman’s bivariate correlation analyses were employed to investigate the associations between the FAR and baPWV and arterial stiffness in the overall population and subgroups of elderly men, non-elderly men, elderly women, and non-elderly women, respectively. To investigate the independent relationships between the FAR level and the baPWV level and the independent relationships between the FAR level and arterial stiffness, multiple linear regression analyses and multivariable logistic regression analyses were constructed. Receiver operating characteristic (ROC) analysis was also carried out to examine the FAR levels’ capacity to detect arterial stiffness.

## Results

### Basic characteristics

In total, 413 T2D patients were enrolled in the study. The mean age was 52.56 ± 11.53 years, 60.8% of the participants were male, and 18.6% of the participants had arterial stiffness. The clinical characteristics of the four subgroups are presented in Table 1 according to the FAR quartiles. The proportion of arterial stiffness and baPWV level both significantly increased (*p* < .001) along with the gradual rise in the FAR quartiles. Age, the duration of diabetes, the utilization of acarbose, triglyceride (TG) level, albumin level, fibrinogen level, hemoglobin level, neutrophil percentage, platelet count, and neutrophil/lymphocyte ratio (NLR) level were all significantly different between the four subgroups (*p* < .05). While there were no significant differences in the proportion of males, smoking and drinking status, systolic and diastolic blood pressure, use of additional antihypertensive and antidiabetic treatments, HbA1c level, eGFR level, urinary albumin-to-creatinine ratio (UACR) level, total cholesterol (TC) level, low-density lipoprotein cholesterol (LDL-C) level high-density lipoprotein cholesterol (HDL-C) level, and white blood cells (WBC) count (*p* > .05).

**TABLE 1:** Clinical characteristics of the study participants.

Variables	Q1	Q2	Q3	Q4	*p* value
FAR (mg/g)	52.25(45.97–55.87)	64.31(62.32–67.01)	76.15(72.99–79.35)	98.40(90.50–117.14)	
FAR (range)	<59.44	59.44–68.96	68.96–83.74	>83.74	
*n*	103	104	103	103	
Age (years)	49.69 ± 11.12	52.12 ± 11.24	52.47 ± 11.61	55.99 ± 11.43	.001
Male, *n* (%)	37(35.9)	39(37.5)	39(37.9)	47(45.6)	.484
Diabetic duration (years)	3.0(.0–10.0)	5.0(1.0–10.0)	5.0(1.0–10.0)	6.0(1.0–10.0)	.049
BMI (kg/m^2^)	25.77 ± 3.62	26.06 ± 4.38	26.13 ± 3.96	25.16 ± 3.48	.268
SBP (mmHg)	130.0(120.0–141.0)	134.0(125.5–146.5)	135.0(125.0–149.0)	135.0(123.0–150.0)	.112
Smoking status					.654
Never smoking, *n* (%)	66(64.1)	70(67.3)	64(62.1)	76(73.8)	
Former smoking, *n* (%)	9(8.7)	6(5.8)	9(8.7)	6(5.8)	
Current smoking, *n* (%)	28(27.2)	28(26.9)	30(29.1)	21(20.4)	
Drinking status					.274
Never drinking, *n* (%)	62(60.2)	62(59.6)	63(61.2)	75(72.8)	
Former drinking, *n* (%)	9(8.7)	5(4.8)	8(7.8)	7(6.8)	
Current drinking, *n* (%)	32(31.1)	37(35.6)	32(31.1)	21(20.4)	
DBP (mmHg)	83.87 ± 10.91	85.68 ± 9.65	84.94 ± 12.22	83.33 ± 10.33	.399
Antidiabetic treatments					
Insulin treatment, *n* (%)	17(16.5)	26(25.0)	34(33.0)	25(24.3)	.056
Metformin, *n* (%)	51(49.5)	48(46.2)	48(46.6)	54(52.4)	.789
Acarbose, *n* (%)	3(2.9)	6(5.8)	3(2.9)	14(13.6)	.004
Insulin-secretagogues, *n* (%)	21(20.4)	27(26.0)	30(29.1)	31(30.1)	.385
Insulin-sensitisers, *n* (%)	9(8.7)	14(13.5)	14(13.6)	7(6.8)	.284
DPP-4 inhibitors, *n* (%)	6(5.8)	11(10.6)	9(8.7)	6(5.8)	.499
SGLT-2 inhibitors, *n* (%)	9(8.7)	17(16.3)	11(10.7)	15(14.6)	.330
Antihypertensive treatments					
CCB, *n* (%)	12(11.7)	24(23.1)	21(20.4)	27(26.2)	.058
ARB, *n* (%)	18(17.5)	15(14.4)	17(16.5)	23(22.3)	.496
β-blockers, *n* (%)	5(4.9)	5(4.8)	7(6.8)	5(4.9)	.899
Diuretics, *n* (%)	5(4.9)	9(8.7)	9(8.7)	9(8.7)	.658
Statins medications, *n* (%)	3(2.9)	6(5.8)	8(7.8)	5(4.9)	.477
HbA1c (%)	9.70 ± 2.04	9.57 ± 2.26	9.32 ± 2.17	9.82 ± 2.26	.389
UACR (mg/g)	11.30(6.45–30.30)	13.00(7.40–27.15)	16.30(8.70–50.75)	16.10(8.35–33.55)	.061
eGFR (ml/min/1.73m^2^)	108.32 ± 22.27	107.19 ± 23.93	102.47 ± 24.22	99.66 ± 25.93	.081
TG (mmol/L)	1.79(1.05–3.14)	1.77(1.08–3.13)	1.89(1.17–3.02)	1.34(.99–2.21)	.015
TC (mmol/L)	4.39(3.82–5.07)	4.40(3.74–5.24)	4.43(3.79–5.14)	4.13(3.70–5.14)	.528
HDL-C (mmol/L)	1.10(.93–1.28)	1.10(.93–1.30)	1.10(.93–1.27)	1.11(.91–1.36)	.973
LDL-C (mmol/L)	2.73 ± .87	2.86 ± .91	2.85 ± .83	2.80 ± .94	.689
Alb (g/L)	39.34 ± 3.62	38.86 ± 3.32	37.44 ± 3.15	36.15 ± 3.11	<.001
Fg (mg/L)	1990(1770–2200)	2500(2360–2650)	2860(2650–3020)	3610(3320–4100)	<.001
Hb (g/L)	145.18 ± 16.28	142.99 ± 13.56	141.57 ± 14.68	133.38 ± 21.01	<.001
WBC (*10^9^/L)	6.10(5.10–7.00)	5.90(5.00–7.05)	6.40(4.85–7.45)	6.30(4.73–7.68)	.588
NEU (%)	54.46 ± 8.83	56.97 ± 8.59	57.97 ± 8.94	61.03 ± 10.30	<.001
PLT (*10^9^/L)	196.00(165.25–237.00)	189.00(157.00–238.50)	201.00(166.50–239.00)	216.00(189.25–267.50)	.001
NLR (%)	1.57(1.24–2.02)	1.79(1.34–2.24)	1.80(1.36–2.45)	2.05(1.56–3.22)	<.001
baPWV (cm/s)	1382.0(1244.0–1548.0)	1489.0(1340.0–1747.0)	1534.5(1294.0–1719.3)	1673.0(1403.0–1865.0)	<.001
Arterial stiffness, *n* (%)	7(6.8)	15(14.4)	20(19.4)	35(34.0)	<.001

Normally distributed values in the table are given as the mean ± SD, skewed distributed values are given as the median (25% and 75% interquartiles), and categorical variables are given as frequency (percentage).

FAR, fibrinogen/albumin ratio; BMI, body mass index; SBP/DBP, systolic/diastolic blood pressure; PAD, peripheral artery disease; Insulin-secretagogues insulin secretagogues, Insulin-sensitisers insulin sensitizing agents, DPP-4, inhibitors dipeptidyl peptidase-4 inhibitors, sodium-glucose co-transporter-2, inhibitors SGLT-2, inhibitors; CCB, calcium channel blockers; ARB, angiotensin receptor blockers; HbA1c glycosylated hemoglobin A1c; UACR, urinary albumin-to-creatinine ratio; eGFR, estimated glomerular filtration rate; TG, triglyceride; TC, total cholesterol; HDL-C, high-density lipoprotein cholesterol; LDL-C, low-density lipoprotein cholesterol, Alb albumin, Fg fibrinogen, Hb hemoglobin; WBC, white blood cells; NEU, neutrophil percentage; PLT, platelets; NLR, neutrophil/lymphocyte ratio, baPWV, brachial-ankle pulse wave velocity.

### Relationships between the FAR and clinical parameters in patients with T2D

As shown in Table 2, the FAR had significant positive associations with age, duration of diabetes, UACR level, neutrophil percentage, platelet count, and NLR level (r = .226, .161, .115, .245, .165, and .263, respectively; *p* < .001), as well as significant negative associations with eGFR, TG, and hemoglobin level (r = −.153, −.127, and −.318, respectively; *p* < .001).

**TABLE 2:** Relationships between FAR and clinical parameters in patients with T2D.

Variables	*r*	*p* value
Age	.226	<.001
Diabetic duration	.161	.001
BMI	−.074	.135
SBP	.095	.053
DBP	−.031	.534
HbA1c	−.026	.603
UACR	.115	.022
eGFR	−.153	.005
TG	−.127	.010
TC	−.041	.402
HDL-C	.035	.476
LDL-C	−.001	.989
Hb	−.318	<.001
WBC	.024	.637
NEU	.245	<.001
PLT	.165	.001
NLR	.263	<.001

*r* spearman’s correlation coefficient.

### Relationships between the FAR and baPWV and arterial stiffness in patients with T2D

Table 3 shows that the FAR, baPWV, and arterial stiffness were significantly positively associated in the general population and subgroups of elderly men and non-elderly men (*p* < .01), but were not significantly associated in subgroups of elderly and non-elderly women (*p* > .05). The independent association between the FAR and baPWV in male patients with T2D was further investigated using multiple linear regression models, as shown in Table 4. In the fully adjusted model 3, the lnFAR demonstrated a significant and positive association with the lnbaPWV in male T2D patients (β = 0.216, t = 4.077, *p* < 0.001, R2 = 0.609).

**TABLE 3:** Relationships between the FAR and baPWV, the FAR and arterial stiffness.

Variables	Total		Male				Female			
			< 60 years		≥ 60 years		< 60 years		≥ 60 years	
*n*	413		201		50		112		50	
	*R*	*p* value	*r*	*p* value	*r*	*p* value	*R*	*p* value	*r*	*p* value
baPWV	.262	<.001	.268	<.001	.435	.002	.057	.548	.050	.731
Arterial stiffness	.266	<.001	.266	<.001	.418	.003	.103	.278	.032	.826

*r* spearman’s correlation coefficient.

**TABLE 4:** Multiple linear regression models displayed independent associations of the lnFAR with lnbaPWV in male patients with T2D.

Models	B (95% CI)	*β*	*t*	*p*	*R* ^*2*^ for model
Model 0	.206(.142–.271)	.370	6.293	<.001	.137
Model 1	.105(.055–.154)	.188	4.188	<.001	.505
Model 2	.118(.059–.176)	.203	3.947	<.001	.546
Model 3	.125(.064–.185)	.216	4.077	<.001	.609

Model 0: unadjusted model.

Model 1: adjusted for age, diabetic duration, BMI, SBP, DBP.

Model 2: additionally adjusted for HbA1c, eGFR, lipid profiles.

Model 3: additionally adjusted for antidiabetic treatments, antihypertensive treatments, statins medications, smoking status, drinking status.

### Association of the FAR with arterial stiffness in male patients with T2D

The multivariable logistic regression analysis of the correlation between FAR and arterial stiffness in male T2D patients is presented in Table 5. In model 0 without any adjustments, the FAR had a significant association with the presence of arterial stiffness [OR (95% CI), 1.039 (1.024–1.054)]. Even in the model with all of the adjustments, there was still an independent association between the FAR and arterial stiffness [OR (95% CI), 1.075 (1.031–1.120)].

**TABLE 5:** Multivariable logistic regression analysis to identify the association of the FAR with arterial stiffness in male patients with T2D.

Models	*B*	SE	Wald	*p*	OR	95% CI
Model 0	.038	.007	27.152	<.001	1.039	1.024–1.054
Model 1	.044	.010	17.788	<.001	1.045	1.024–1.066
Model 2	.049	.013	14.367	<.001	1.050	1.024–1.077
Model 3	.072	.021	11.448	.001	1.075	1.031–1.120

Model 0: unadjusted model.

Model 1: adjusted for age, diabetic duration, BMI, SBP, DBP.

Model 2: additionally adjusted for HbA1c, eGFR, lipid profiles.

Model 3: additionally adjusted for antidiabetic treatments, antihypertensive treatments, statins medications, smoking status, drinking status.

### The cutoff FAR value to indicate arterial stiffness

**FIGURE 2:**
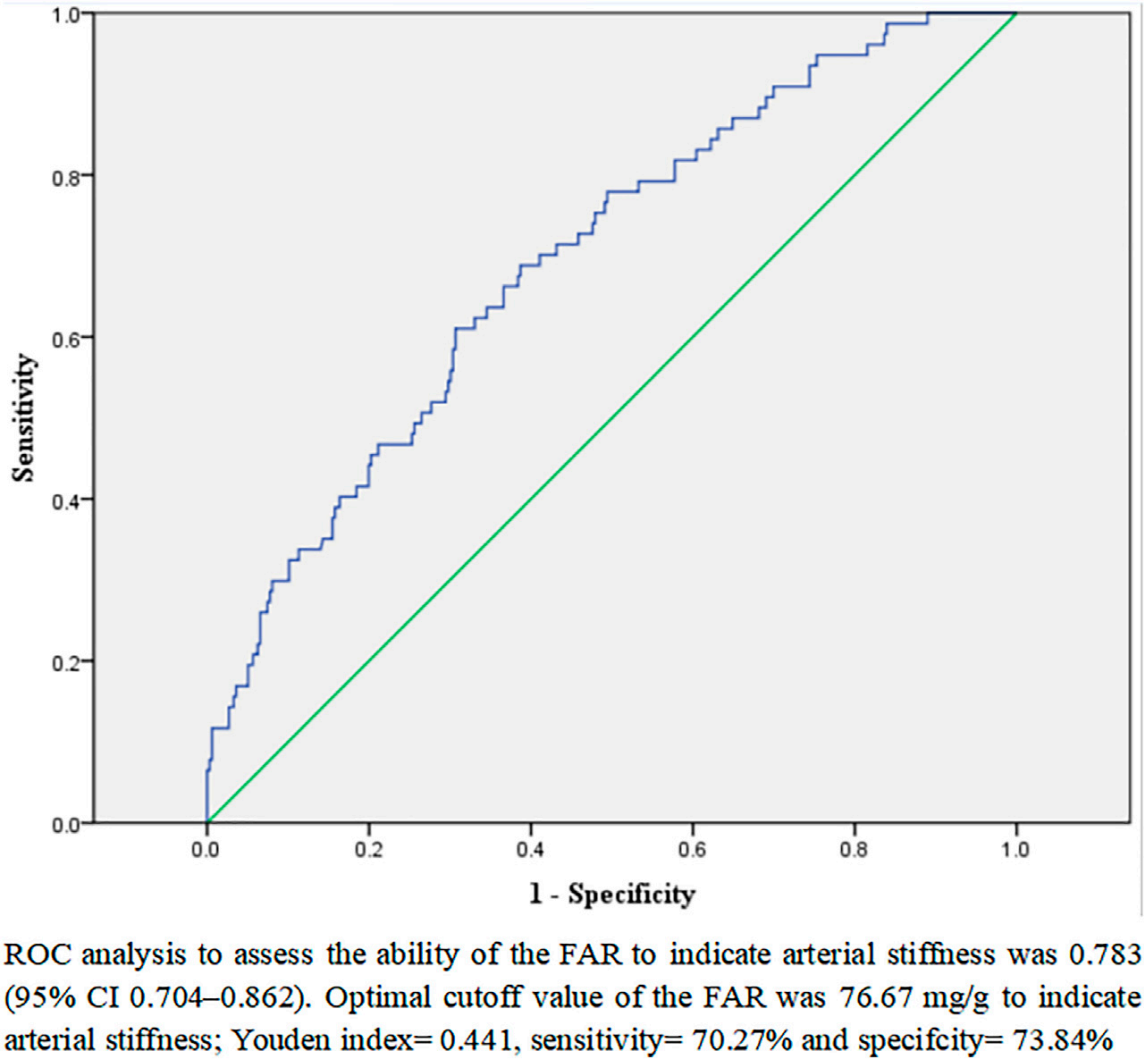
ROC analysis to analyze the ability of the FAR to indicate arterial stiffness in male patients with T2D.

In addition, a ROC analysis was conducted to determine the FAR cutoff value that would indicate the presence of arterial stiffness instances in male T2D patients. As depicted in Figure 2, the optimal FAR cutoff value to indicate arterial stiffness was 76.67 mg/g, with a sensitivity of 70.27%, a specificity of 73.84%, and an area under the curve of .783 (95% CI 0.704–.862).

## Discussion

In the present investigation, the association between FAR and arterial stiffness as determined by baPWV in T2D patients was investigated. In comparison to female T2D patients, male T2D patients had a positive association between the FAR and baPWV and the prevalence of arterial stiffness.

Arterial stiffness can increase cardiac afterload, causing cardiac hypertrophy and left ventricular dysfunction (Tomiyama and Shiina, 2020), as well as amplifying pulse pressure, reducing coronary perfusion, and causing brain microvascular injury and other target organ damage (Vlachopoulos et al., 2015). Both of these factors ultimately contribute to a poor prognosis in diabetic and non-diabetic patients. An increase in baPWV of 1 SD was linked to a 1.41-fold rise in the risk of cardiovascular events, according to a meta-analysis of 15 clinical studies (Sang et al., 2021). In patients with CVD who are controlling for traditional risk factors, improvement of baPWV provides an additional protective impact (Nakamura et al., 2021). In the current investigation, FAR was found to be independently correlated with arterial stiffness and may thus be significantly associated with cardiovascular prognosis in male T2D patients. The SYNTAX score, derived from the lesion angiographic scoring system, is frequently used to quantify the severity of CVD (Girasis et al., 2011). Multiple investigations have confirmed the association between FAR and SYNTAX scores in AMI patients (Erdoğan et al., 2016; Erdoğan et al., 2021). In addition, the relationships between FAR and the severity of coronary arterial calcification, the Gensini score, and the number of diseased coronary arteries were identified (Duan et al., 2021; Zhu et al., 2022). In a large cohort analysis with a 5-year follow-up, the combination of a high FAR level and diabetes predicted a worse prognosis in patients receiving the percutaneous coronary intervention (PCI) (Wang et al., 2022). Thus, these findings suggest that FAR may be useful in assessing the risk of CVD as well as determining the severity and predicting the prognosis of CVD in T2D patients.

Arterial stiffness is caused by a combination of variables such as an imbalance in elastin collagen ratio, oxidative stress, chronic inflammation, and so on, and diabetes can accelerate this process (Lee et al., 2022). Numerous investigations have revealed that arterial stiffness is closely correlated with T2D’s essential components, including insulin resistance (IR) (Lee et al., 2018), glucose fluctuations (Wakasugi et al., 2021), and chronic hyperglycemia (Katakami et al., 2020). In turn, arterial stiffness can induce IR (Chirinos, 2020) and islet damage (Tian et al., 2022) by damaging capillaries, ultimately increasing glucose metabolism issues. The relationship between the two may be inflammation and hemodynamic alterations (Sharif et al., 2021).

In a cross-sectional examination of risk factors for diabetic kidney disease (DKD) in T2D patients, FAR was significantly found to be positively associated with neutrophil count and NLR, leading researchers to hypothesize that FAR may be substantially associated with inflammation and consequently DKD (Wang et al., 2021). Similarly, the present investigation found a significant positive association between FAR and neutrophil percentage and NLR, suggesting that inflammation may function as a link between FAR and arterial stiffness. A higher FAR indicates a lower level of albumin or a higher level of fibrinogen. Hypoalbuminemia is a biomarker for inflammatory load in the body because inflammation can lower the rate of albumin production and increase albumin catabolism (Cesari et al., 2003). Additionally, albumin can prevent inflammation-induced endothelial cell apoptosis by inhibiting cell adhesion molecule expression (Albert et al., 2007). Therefore, the independent relationship between arterial stiffness and FAR may be partially explained by inflammation.

The relationship between FAR and platelet count, a critical component of the coagulation system, was found to be statistically significant and positive in this study. Platelet, the primary cause of coronary thrombosis and atherosclerosis, is an essential target for the management of coronary heart disease (Khodadi, 2020). Albumin has been shown to suppress platelet activation and aggregation, whereas activated platelets can cause coronary artery contraction and worsen myocardial ischemia (Sweetnam et al., 1996). Albumin can also indirectly control platelet aggregation by influencing prostaglandin bioavailability (Garcia-Martinez et al., 2013). In addition, fibrinogen can be converted to water-insoluble fibrin by thrombin, but hypoalbuminemia can block the physiological fibrinolytic system, hence limiting spontaneous thrombus dissolution (De Sain-van der Velden et al., 2000). As a result, FAR may be related to arterial stiffness and reflect changes in the hemodynamic state of T2D patients.

The relationships between arterial stiffness and the FAR in T2D patients were shown to differ by gender in this study. Numerous investigations have conclusively shown that the clinical features and sex-specific processes underlying arterial stiffness in males and females were different (DuPont et al., 2019). In a cross-sectional investigation of T2D patients, there was a higher correlation between arterial stiffness and surrogate indicators of insulin resistance in women than in men (Nakagomi et al., 2020). The findings of the present investigation may be partially supported by these studies.

The following are the study’s limitations, which cannot be avoided. First, the limitation of cross-sectional studies prevents the demonstration of a coincidental relationship between FAR and arterial stiffness. Second, due to a lack of data, it was impossible to account for confounding variables, including detailed alcohol and tobacco consumption and daily activity levels. Finally, all of the participants in this study were Chinese, which may restrict the generalizability of the findings.

In conclusion, the FAR level was positively and independently associated with baPWV and arterial stiffness in male T2D patients. When considered in combination with other investigations, FAR has the potential to assist in the better management of cardiovascular problems in patients with T2D, particularly male patients.

## Data Availability

The raw data supporting the conclusions of this article will be made available by the authors, without undue reservation.
